# Our Insane

**Published:** 1884-02

**Authors:** S. V. Clevenger

**Affiliations:** Special Pathologist Cook County Hospital for the Insane


### ARTICLE VI.

OUR INSANE. A Lecture Delivered Before the Chicago Philosophical Society, January 19, 1884. By S. V. CLEVENGER, M.D., Special Pathologist Cook County Hospital for the Insane.

Volumes have been and will be written concerning what has been done, is being done, and will be done for the insane, and anything like an attempt at an exhaustive review within our present limits must fall short of the vastness of the subjects involved. A purely sociological view of alienistic matters would require inclusion of allied considerations the scope of which would drive us further from the matter in hand. Let us, then, look at the insanity about us from the standpoint of the alienist, who comes most in direct contact with our insane and the problems suggested by their existence. Retrospective and prospective generalizations are afforded from this vantage ground as follows:

1. Scientific inquiry into the causes, phenomena, and treatment of insanity has resulted in an increased number of recoveries, more humane treatment of the insane, and enlightenment as to the prevention of insanity.

2. We are indebted to European research for the greater part of our knowledge of insanity as a disease, and comparatively little has been done in America to forward psychiatrical studies.

3. Popular conservatism due to lack of information on these subjects is the cause of the national apathy in this respect, but eventually an activity in the prosecution of these studies will prevail in the United States unsurpassed in any other part of the world.

Insanity is the inability to be impressed and to conceive quantatively and qualitatively enough to guide actions in harmony with the individual's age, circumstances and surroundings, and the failure to properly coördinate impressions and frame logical conclusions and actions, except when occurring in the course of sleep, trance, somnambulism, coma, or simple epilepsy, hysteria and chorea, or during ordinary mental abstraction.

But the futility of making precise relative definitions is well known. No matter how thoroughly the pathology of disease of any kind may be known, disease itself is incapable of definition. The mathematician's definition of a point is an absurd one, owing to our inability to determine the boundaries of the finite. Where mentality begins and ends must be conditioned, and medically it is sufficient that the brain is the accepted organ of the mind, and that insanity is a manifestation of a brain disorder. The definition attempted above is modified from Spitzka's, and doubtless not improved. Reference to hallucinations is omitted, for they may exist without insanity. Germany, Italy and France have dropped definition discussions. England and America, in their medico-legal evolution, have perpetuated them, as it is found a convenient stumbling-block in the way of medical testimony.

Prevalent ideas of insanity are vague ones, and biased as are all sociological conceptions. One who visits an insane asylum for the first time finds little therein as he had mentally pictured it. Many of the patients are at liberty to come and go at will, some being merely paroled on their honor not to leave the grounds. You may observe gangs of insane workmen for weeks and fail to diseover anything indicating want of ordinary intelligence among them. Female patients occupied in the sewing room seem intent upon their work, though an occasional silly act or expression may escape them. In most wards, as a rule, there is quiet and order, the inmates pace to and fro, or are seated about apparently absorbed in thought, the cases requiring more control are gathered together in one part of the building, and here the ideal madman may be seen with hair erect, eyes protruding, flushed, angry countenance, demonstrative and boisterous; but he may not be so dangerous to others as the quiet maniac who watches his opportunity to catch you off your guard. Every degree of physical condition is observable, from the robust to the weakly. Some are confined to bed by sickness, others appear to possess the greatest vigor. Strength and ferocity sometimes exist in the same individual, and as may be imagined, it forms a disgreeable combination. The weakly are as apt to be troublesome, and usually supplement their lack of muscle by craftiness. Destructiveness is a prominent trait in a large number of cases. Walls are defaced, windows and furniture broken, bedding and clothing torn into shreds, regularly, day after day, or night after night by some, and only at long intervals by others. The tendency of repeated attacks of furor is eventually toward terminal dementia, "that tomb of the mind whence no errant intellect ever returns." In this stage the lowest automatic movements of the body are alone performed, the individual breathes, eats and sleeps, but must be led and fed, nothing can arouse him from his stupor, and death approaches all too slowly. One of the most noticeable forms of insanity met with in asylums is known as melancholia agitata, wherein there is much weeping, verbigeration and wringing of hands, with other actions indicative of distracted grief, but a more horrible form of melancholia exists which would escape your notice altogether, owing to the listless apathy of the sufferer. Melancholia attonita, or "thunder-struck melancholia," is a mental condition of great torture. Those who have emerged from it with sound minds state that they have seen their relatives and friends torn limb from limb, and that the food offered them was soaked in human blood. Every imaginable fiendishness was enacted before their eyes with the accompaniments of groans, shrieks, appeals for help and other frightful sounds. These phantasms are terribly real to those who experience them, and resemble vivid nightmares.

When the furibund condition is not present, the chronic maniac is not apparently distressed by his hallucinations. He accepts his vagaries as real, and confuses the purely subjective with the really objective scenes. Skeletons, ghosts, and other hideous forms mingle with the actual personalities about. Divinities, saints, angels, and devils float to him from the sky. Voices, pleasant and unpleasant, follow him, and frequently in obedience to their commands he will do violence. Thus, upon one occasion a negro at the County Asylum, while looking from his window saw God in a chariot, who commanded him to break his way out. An hour thereafter the corridor looked as though a tornado had swept it. The exalted emotional condition of simple mania may never reach the explosiveness of furor, the entire life of a hypomaniac may be one day dream. Everything seems beautiful to him ; trees, fountains, flowers, birds, gorgeous surroundings, artificial as well as natural, abound in this phase. Everybody and thing seems pleasant, no matter what the surroundings may be. The heart is happy, the mind is blissfully contented. Something similar obtains at times in the delusions of grandeur attending paretic dementia. Millions of dollars, diamonds, ships, railways and great enterprises, or intellectual achievements are claimed by him. Often he is a great magnate, general, or even deity. This is an incurable form of madness, with an average duration of three years, during which the sense of touch is so blunted that injuries may be sustained without consciousness of them, and by paretic dements the invariable declaration is made that they feel perfectly well, or never felt better in their lives.

All know the frequency with which homicidal attempts are made by the insane, and many an ugly scar across the throat testifies to an attempt at suicide previous to admission. Every conceivable shade and variety of the many forms of insanity exist and frequently blend with each other confusingly, but in the main Spitzka's classification is justified by well-defined cases, such as simple mania, simple melancholia, katatonia, transitory frenzy, stuporous insanity, primary and secondary confusional insanity, primary deterioration, terminal dementia, senile dementia, hebephrenia, paretic dementia, luetic dementia, dementia from coarse brain disease, delirium grave, alcoholic, hysterical, epileptic and periodical insanities, idiocy, imbecility, cretinism, monomania, traumatic, choreic, post febrile, rheumatic, gouty, phthisical, sympathetic and pellagrous insanities.

All forms of insanity then known were included by Hippocrates in mania, melancholia and dementia.

In the eleventh century Gheel, in Belgium, became a resort for the insane, brought thither by a prevalent superstition that divine interposition in their behalf could be had there, and today, at this town, a large population is engaged in caring for lunatics on a peculiar system, one or two patients being allotted to a family, under the supervision of Dr. Peeters. As sociological investigations have but recently begun, and history in the past afforded us mainly personal gossip about princes, the ideas concerning insanity in olden times have been but imperfectly gathered, but enough is known to convince us that ancient tribes were not agreed as to the causes or treatment of lunacy, but in the main regarded it as evidence of the possession of a benign or malign spirit. Records of devils exorcised and of especial protection, even honors, accorded lunatics, show something of the way in which they were regarded. Court fools were frequently idiotic, monomaniacal, or imbecile, and from the manner in which existing savage, barbarious or semi-civilized people treat their insane, we may ascertain ancient beliefs referring to them handed down to the present through ages by these non-progressives. Western

American Indians often abandon their senile dements as well as other infirm and aged relatives, to the wolves, and to die of hunger, thirst, and cold. Idiots are destroyed by them, but imbeciles and other insane are regarded as especially favored by the Great Spirit, and allowed to do as they please. Among Mohammedans generally great reverence is shown certain forms of insanity, amounting almost to worship. Nations in their evolution have oscillated between deification of the insane on the one hand, and extreme cruelty towards them on the other, and it is not so long ago that they were burned alive and drowned as witches or possessed, in our own country. There has been a great advance made in this century in our treatment of the insane. We have to congratulate ourselves that we no longer beat and chain them, we have ceased to treat them as brutes and as possessed by devils; they are now only regarded in law as criminals.

Chiarurgi, in Italy, and Pinel, in France, inaugurated the cessation of cruelties, and in 1839 Dr. John Conolly, of England, abolished all mechanical restraint. He wrote, "Indeed it would seem as if at the period from the middle to near the end of the last century, the superintendents of the insane had become frantic in cruelty from the impunity with which their despotism was attended. The first principle in the treatment of lunatics was fear, and the best means of producing fear was said to be punishment, and the best mode of punishment, stripes. By degrees restraint became more and more severe, and torture more and more ingenious. An unsuspecting patient was sometimes induced to walk across a treacherous floor, it gave way and he fell into a bath of surprise, and was there half drowned and half frightened to death. In some continental asylums the patients were chained in a well and the water was allowed gradually to ascend, in order to terrify the patient with the prospect of inevitable death. The melancholy were tossed about in machines constructed for the purpose, to induce them to take a natural interest in the affairs of life." At Bethlem, Conolly saw ten females chained by arms or legs to the wall, clad in rags and filthy. He details many revolting scenes such as this, and concludes with the statement that the patient, who has created indescribable turmoil at home, when taken to an asylum conducted on good principles, often becomes quite an altered person, disturbing nobody and behaving peaceably. Such sudden improvements certainly almost exceed belief, but the instances are not even rare. Every physician conversant with insanity has witnessed these marvelous metamorphoses many times. The European asylums underwent great changes for the better under Conolly and the Tuke family early in this century, but in the report of the Commissioners (Quincy, Hitchcock and Storer) on Insanity, to the Legislature of Massachusetts, (Pub. Doc.. 1864, Senate, No. 72) almost similar brutalities were unearthed, and the *American Journal of Insanity* for January, 1865, details more horrors as occurring in two other States in the Union. While America has, in many respects, led the world to grander conceptions of liberty and humanity, our country has lagged behind the monarchical governments in the matter of humanity to the insane. Reform movements have sprung up in the Union, the most noted being headed by Miss Chevalier, an energetic, enthusiastic lady, who has done more good than many ponderous missionary boards. Maudsley sums up the present non-restraint measures as follows :

"Its principle is to avoid a meddlesome interference; to make all the surroundings of the poor lunatic as tranquil, as orderly, as gentle as may be consistent with his proper care; to counteract the commotion in him by the absence of commotion in what is around him. The lunatic cannot any more than the sane person resist the steady influence of his surroundings; he assimilates them unconsciously, and they modify his character for good or for evil."

Herbert Spencer, who is a great advocate of kindness in the treatment of children, in "Social Statics" extends his views to treatment of the insane. "Let those who have no faith in any instrumentalities for the rule of human beings, save the stern will and the strong hand, enter the Hanwell Asylum for the Insane. Let all self-styled practical men, who, in their semi-savage theories, shower sarcasm upon the movements for peace, go and witness, to their confusion, how a thousand lunatics can be managed without the use of force. Let these sneerers at 'sentimentalisms' reflect on the horrors of mad-houses as they used to be, where was weeping and wailing and gnashing of teeth, where chains clanked dismally, and where the silence of the night was rent by shrieks that made the belated passer by hurry on shudderingly ; let them contrast with these horrors the calmness, the contentment, the tractability, the improved health of mind and body, and the not unfrequent recoveries, that have followed the abandonment of the strait-jacket regime, and then let them blush for their creed." Thanks to an awakened public interest, the non-restraint system is fast growing in favor all over this country, and asylums are beginning to vie with each other in making the surroundings of their unfortunate inmates pleasanter. Skillfulness is supplanting brutality in their control, and better results follow.

Intrinsic forces, such as a gradually introduced better class of managers ; and extrinsic forces, such as public opinion, investigations, and the growing inclination of the people to examine public institutions of the kind for themselves, are surely elevating matters. Wherever a pride is taken in bettering the condition of patients, visitors are sure to find pleasant receptions, and the more intelligent classes are especially welcome. Dr. Spray, the superintendent of our county asylum, extends to every one of you a standing invitation to inspect everything about that building, and it is your duty, as citizens, to acquaint yourselves with the status of such institutions.

Now, while the humanizing influences have worked their reforms, and trans-Atlantic examples have been followed to the extent of stopping brutalities, while crimes of commission are seldom heard of, crimes of omission prevail. There is much to be done before psychiatry in America will have approximated the high standing it enjoys in Germany, France, and England. Dr. Tilden, the superintendent of the California State Asylum, embodies in his report words that should be well weighed by legislators and the public of our entire country.

Speaking of his establishment, Dr. Tilden says: "Its beautiful edifice, its well cultivated yards and gardens, its wholesome food, its comfortable clothing, its scrupulously clean halls, rooms, beds, and bedding, its excellent police regulations, combine in making a prison of the first class ; and if such was the original purpose, I see not how it could have been more admirably accomplished. If, however, in creating a charity so munificent, so noble, it was intended to establish an asylum with hospital appliances for the *cure*, as well as the care and safe keeping of the insane, I am free to say it is, in my opinion, a most signal failure. If there is any difference between it and a well conducted State prison, it is in favor of the latter, from the fact that means of employment are provided for its inmates, while the inmates of the asylum spend their days in idleness. It will hardly be contended, I think, that our newspapers and a little gymnasium, with a solitary swing in the female department, can give the asylum of California a claim to the character of a curative institution."

It is the avowed desire of the American Association of Superintendents of Hospitals for the Insane, to introduce improved curative measures into asylums as rapidly as possible, and to establish hospitals for the insane, instead of the old prison asylums.

Herbert Spencer points to the fact that all institutions grow, evolve; they cannot spring into existence. What is to be done must come about gradually, and fifty years hence, our present asylum jails will be considered only as improvements on the ancient slaughter-houses in which the insane were penned up.

The duties of a superintendent of an asylum are similar to those of the landlord of a giant hotel, and in addition, he is the principal medical officer, with often but one or two assistants, himself having immediate charge of a number of wards. With the multitude of details requiring supervision in great caravanseries, imagine Messrs. Palmer or Drake during a cholera epidemic, with their houses filled with sick and dying, compelled to act as medical supervisors. But, says everyone, this is easily enough remedied. Let a business man look after matters pertaining to the stewardship of the asylum, and allow the physicians their legitimate field and nothing more. The experience has been so often repeated, it is coming to be almost common knowledge that this system has invariably failed. There is not a movement; not an ounce of supplies purchased; not a turn of a wheel, but has direct reference to the welfare of the patients in the asylum. Who can possibly be so well informed as to the needs of the establishment as the superintendent, who so well able to direct the movements of men, machinery, and supplies about the place as he? Introduce your business agent with his ideas, in nine cases out of ten antagonistic to the medical superintendent's, simply because he has not the same matters at heart, the same problems to solve, nor the least appreciation of whys and wherefores, and there is constant necessity for endless explanations, arguments, appeals, etc., whenever trifling, to say nothing of important, matters are at stake; or, what is more apt to take place, there are clashings of authority, and the disgusting bickerings of a house divided against itself. Every alienist of note has given his opinion that the superintendent of an asylum should be supreme in command in the asylum as to all persons and things, and that no one disloyal to him, be he officer or employe, should be allowed to remain an instant.

Too often it is the case that petty strife abounds in these places, and, as everywhere else in the world, intriguing for place, with its plexus of lies, recriminations, and plots occur. The lives of nearly everyone about in that event are embittered, and at best it is a difficult matter to reconcile the heterogeneous ways of thinking encountered in large asylums. A superintendent has to be naturally a large-minded man to escape the effects of constant contact with the littlenesses of others.

The individuality of a superintendent will tinge the entire place. If he is small, small will be his servants. If he is lazy or cruel, so will they be. If his views are large, and he possess a compassionate heart, and is determined to do his utmost for his patients, that spirit will pervade the house, from the second medical officer to the scullion.

There is no escape for the superintendent from the oversight of details, but the anxiety and time devoted to them could be transferred to more purely medical affairs by affording him all latitude in the employment of faithful assistants and the elimination by prompt discharge of the unfaithful. All this presupposes the chief officer to be himself well fitted for the place. The frequent changes made in these positions, and the selection of inexperienced persons, produces great confusion and invariable retrogression. In Europe, the appointment of a superintendent without previous asylum experience is unheard of. Positions are secure from other than medical and moral considerations, and a liberty of action and opinion is engendered among physicians directly caring for the insane unknown in our country. Upon superannuation, after faithful service, these officers are pensioned by their governments. Where the tenure of office is uncertain, there is little incentive to honest work, and great temptations for intriguing on the part of outsiders to dispossess those in office, not from any medical, scientific, or philanthropic animus, but from mere place-hunting greed. Who can work with the calmness and peace of mind necessary to carry on complex professional duties over a volcano which any minute may explode into a financial wreck, utterly regardless of the years of study and the fortune one may have expended in fitting oneself for an especial field.

The prime causes of all these embarrassments in the way of psychiatry are mainly the crude ideas among the people, as exhibited in their laws regulating the arrest and custody of lunatics. The strangely erroneous notions of insanity extant originated and perpetuate a very defective jurisprudence. The insane must either be held responsible for his actions, or he must not be. If not, then the question arises, how much insanity is required to exempt one from the consequences of having committed a crime? The ability to distinguish right from wrong is no guide as to responsibility, for an epileptic may be at one time in full possession of his faculties, and at another absolutely unconscious of what he does, and epileptics afford us the most terrible form of mental alienation. The literature of this subject is abundant, but we may only glance at portions. The English law seems fair enough which places responsibility, if at the time of committing the offense the accused knew right from wrong, but in the determination of this point juries will hang the more insane, and let the less insane escape, often acquitting one and condemning another, both having the same form of insanity and degree of responsibility, and even the knowledge of right and wrong does not bear scientific scrutiny as a means of judging responsibility.

In the case of *State vs. Pike* Chief Justice Perley, of New Hampshire, instructed the jury that they should return a verdict of not guilty, "if the killing was the offspring of mental disease in the defendant, that neither delusion, nor knowledge of right or wrong, nor design, or cunning in planning and executing the killing, and in escaping or avoiding detection, nor ability to recognize acquaintance, or to labor or transact business, or manage affairs, is, as a matter of law, a test of mental disease; but that all symptoms and all tests of mental disease are purely matters of fact to be determined by the jury." "A striking and conspicuous want of success," said Judge Doe in the same case, "has attended the efforts made to adjust the legal relations of mental disease. It was for a long time supposed that men, however insane, if they knew an act to be wrong, could refrain from doing it. But whether that suspicion is correct or not is a pure question of fact, in other words a medical supposition—in other words a medical theory. Whether it originated in the medical or any other profession, or in the general notions of mankind, is immaterial. It is as medical in its nature as the opposite theory. The knowledge test in all its forms and the delusion test are medical theories introduced in immature stages of science in the dim light of earlier times, and subsequently, upon more extensive observations and more critical examinations, repudiated by the medical profession. But legal tribunals have claimed these tests as immutable principles of law, and have fancied they were abundantly vindicated by a sweeping denunciation of medical theories, warning that this aggressive defense was an irresistible assault on their own position. In this manner opinions purely medical and pathological in their character, relating entirely to questions of fact and full of errors, as medical experts now testify, passed into books of law, and acquired the force of judicial decisions. Defective medical theories usurped the position of common law principles. Whether the old or the new medical theories are correct, is a question of fact for the jury. It is not the business of the court to know whether any of them are correct. The law does not change with every advance of science, nor does it maintain a fantastic consistency by adhering to medical mistakes which science has corrected. The legal principle, however much it may formerly have been obscured by pathological darkness and confusion, is that a product of mental disease is not a contract, a will, or a crime. It is often difficult to ascertain whether an individual has a mental disease, and whether an act was the product of that disease, but these difficulties arise from the nature of the facts to be investigated, and not from the law, they are practical difficulties to be solved by the jury, and not legal difficulties for the court."

In *State vs. Jones*, Judge Ladd, on the right and wrong test of responsibility, claimed that to be "an interference with the province of the jury and the enunciation of a proposition which in its essence is not law, and which could not in any view safely be given to the jury as a rule for their guidance, because, for aught we can know, it may be false in fact." These opinions are quoted as embodying most advanced views. Witchcraft was dealt with by the law much as insanity is to-day. The jury were given erroneous instructions on matters of fact under the name of law. Lord Hale decided that there were such creatures as witches, for the Scriptures had affirmed as much, the wisdom of all nations had provided laws against such persons, which is an argument of their confidence of such a crime. But the enlightened opinion of the country made the witch he condemned one of the last to be executed in England. As with witchcraft, so to-day with insanity, the judge instructs the jury wrongly on matters of fact, and the jury finds a verdict accordingly."

Judge Doe further said, "If the tests of insanity are matters of law, the practice of allowing experts to testify what they are should be discontinued; if they are matters of fact. the judge should no longer testify without being sworn as a witness and showing himself qualified to testify as an expert."

In *Boardman vs. Woodman* the same authority states, "If it is necessary that the law should entertain a single medical opinion concerning a single disease, it is not necessary that that opinion should be a cast-off theory of physicians of a former generation."

Expert testimony is another aggravating feature of law courts. As long as men are as they are, physicians as well as others will, for the sake of the fee, and without the least preparation or fitness, enter the courts as experts and pass upon subjects the nature, literature and facts of which they are ignorant; and again, men of special training will prostitute their calling by testifying with partisan zeal, suppressing the truth as they know it, and misleading the jury as to facts. This state of things should not be countenanced. The expert may coach a lawyer, but should not appear as a partisan. He should appear as an expert to tell the truth as he knows it, regardless of how it may affect the case. If his honest convictions are with one side or the other, he may be engaged by defendant or plaintiff; but having the confidential communications of one side, he may not subsequently appear against that side. And the presentation of the hypothetical case is not a correct method of eliciting the truth, for it is impossible to embody all the details necessary to a full understanding of the case by such means. Dr. Ray thought the expert should be asked, "Do you give your time and attention continuously to a particular branch of medicine, and is that mental, or psychological medicine? and that opinions should be given in writing, and read to the jury without oral examination. It would then be deliberately prepared, its explanations well considered, and its full force and bearings clearly discerned. It would go to the jury on its own merits, no advantage being gained by either party by the superior adroitness of counsel in embarrassing the witness and pushing his statement to a false or ridiculous conclusion." The animus of the expert, with his dishonesty or incompetence, would thus be disclosed.

The Illinois law provides that the sheriff, jailer or other suitable person may detain persons until trial. Both jailing and the trial by jury of the insane are processes unworthy a civilized age and people. The Board of State Charities takes proper ground in this matter, in its last report, as follows:

"Does not the uncertain condition in Illinois under our law demand a return to the common sense law, with modifications, once existing in this State, which virtually treated an insane person as mentally sick, and did not require him to be treated as a criminal and be tried by a jury? What good has been effected by the change in the law? We maintain that no good has been done, and that serious questions arise, clogging the individual's future, and also attaching more of a stigma, if such it be, of insanity by the finding of a jury. Why not leave the matter, as in many of our States in the United States and as in England, to be dealt with as a scientific, professional question for the medical man and pathologist, and not for the finding by a verdict of jurors, based on slight evidence? Is it essential to liberty and to the maintaining of personal freedon from undue restraint, that the law should exist in its present form? A writ of *habeas corpus* will always lie as a writ of right to inquire into the cause of the detention of any party in an hospital for the sane or insane. It is believed by many that our present jury law was superinduced by undue excitement growing out of one case, which was by no means a clear case of misapplication of the rigor of the law. It is required that the near relatives (and when none exist, then a respectable person of the county) must petition for the trial of the person's sanity or insanity, and it is obvious from the law that the proceeding is for the welfare of the individual supposed to be insane. It is not a criminal charge, and yet you treat the matter with the formality of a charge or trial for crime; in place of having a commission or board of physicians, you try the person and render a verdict, from which you provide no escape by his individual act that would be legal."

The *Alienist and Neurologist*, in commenting on this, adds: "Let the law pile high the penalties for false certificates of insanity, and searchingly inquire as to the qualifications and responsibility of those who may sign them, but save the poor lunatic from the uncertain chances of speedy treatment through a petit jury trial."

The time lost during the jail incarceration, which might have been spent under hospital treatment, in many cases confirms the insanity for life, and every alienist knows the importance of prompt treatment in recently developed cases; frequently all that can be done is at the inception of the malady. Many of the insane dwell persistently upon these court proceedings, and their delusions are tinged with them.

Those with delusions of persecution frequently find confirmation of their fears in these trials and commitments, and some talk of but little else. Male case No. 212 and female case No. 263, at the County Asylum, are instances.

Investigations are being made to ascertain if insanity is increasing. In the present state of statistical information much can be said for and against such a conclusion. The apparent increase may be due to more insane being registered now than formerly. In 1859, in England, the rate was 1 to 540; in 1876, 1 to 375. In the United States the census returns numbered 44,148 insane in 1870, the proportion being 1 insane to 953 of the entire population. California's proportion being 1 to 484. At the same time in England, it was 1 to 403; in France, 600; Scotland, 336; Ireland, 302; and Russia, 450. Farr states the rates for upper and middle classes in England are 1 to 484, and lower classes 1 to 353. While asylums show more females than males under treatment at a given time, there is actually more insanity among men everywhere than among women. The apparent anomaly is caused by the females being more capable of enduring an indoor life than the males, a larger part of whom either recover or die sooner than females, and consequently are present in less proportion.

As to causes of insanity, Dr. Funkelburg, of the Russian Public Health Commission, claims that in that country lack of intellectual culture, insufficient food, unhealthy dwellings, are causes, but abuse of alcoholic liquors sends 20 per cent. to asylums and 40 per cent. to jails.

Lord Shaftesbury, who was chief of the lunacy commission for fifty years, said that 50 per cent. of insanity in England was caused by intemperance. According to Lunier, 50 per cent. of the idiots and imbeciles in Europe had notoriously drunken parents. Michét figures hereditary influences as predisposing to one-half of all insane cases. Guslain claims heredity operative in 30 per cent. of cases, and Anstie ascribes the origin of heredity to want of education, mental vacuity, and excesses, particularly alcoholic. A good instance of the misery entailed by degraded ancestry is afforded by the Jukes family, of New York. Margaret Jukes, in six generations, had 709 descendants, all of whom were thieves and murderers or idiots. Exciting causes in one generation become predisposing in the next. Dr. Page, of the Connecticut (Hartford) Retreat, analyzed 2,333 cases, and found one-half due to causes largely under the control of man. Hereditary tendencies were at the bottom of most cases. the bad habits of parents becoming diseased conditions in the children. Head injuries and sunstrokes are prolific sources of insanities. Diseases such a scarlatina, meningitis, small-pox, rheumatism, gout, lues, epilepsy, chorea, hysteria, predispose to and directly cause insanity. Pellagrous insanity is a form produced by eating diseased corn. Morphine, chloral, and other poisons sometimes cause loss of mentality. Emotionalism, such as is engendered by too much novel reading and failure to cultivate reasoning faculties, is a cause. Consanguineous marriages often predispose offspring to weak mental condition. Change of environment, domestic troubles and griefs, roughly estimated, cause 12 to 15 per cent., but preëxisting mental instability may be supposed in many such cases. The two extremes of mental stagnation with sensual degradation upon the one hand,and gloomy fanaticism on the other, and dwelling too long and intently upon religious questions, especially when presented in narrow and exclusive forms, drive to despair or perilous exaltation of the feelings.

It may be conceded that there is an absolute necessity for taking care of the insane, for society and individuals would suffer for want of protection against them if not for them. The altruism of this public charity is not so evident as its egoism. The assumed function of an asylum is for the care, and, if possible, cure of the insane; the State is not supposed to entomb every one admitted as hopelessly insane.

Thirty years ago, Dr. Forbes Winslow inveighed against the dogma pervading all grades of society, "Once insane, always insane." It is an enormous assumption, one of the lying old saws, which among unreflecting people is chargeable with having done much harm. Many patients recover from insanity never again to become insane. Many more are so far improved as to be of use in the care of other insane. Were this not the case the expenses of maintenance would be immensely greater than they are, for the work done by inmates is an important feature of the asylum. Are we justified in doing anything at all for the insane? Should they not be allowed to perish as rapidly as possible, without effort in their behalf? are questions a common humanity has answered by the establishment of asylums, and having thus answered, the care of these unfortunates must be advanced in keeping with the progress of knowledge, for neglect in part would justify neglect in toto, which is equivalent to asserting the right of the State to destroy its lunatics. If a charity is worth carrying out at all, it is worth doing well. The falsehood that no one recovers from insanity has done its evil work, let the knowledge that many recover, and that the advance of medical science has rescued a still larger number, do its good work. If the plea is made that by this means hereditary taint is propagated, it may be shown that it as frequently prevents its propagation, and even granting it, failure to provide every reasonable means for cure and care may be extended to ordinary bodily diseases. All hospitals should be converted into tombs, medicine should be dismissed and phthisis, wounds, and every other ailment be allowed to run their course with the fatalistic justification of the Menonnites. No help should be afforded the drowning man, his death will ensure the propagation of a more cautious race. One-half of the world is subject to inherited bodily taint, and an extension of the *laisser faire* of asylum treatment would justify one-half the world in destroying the other half, and destruction is the same in result, whether by direct or indirect means, by neglect or by assault.

Granting that the insane should be cared for, the accusation cannot be made that America does not house, clothe, warm, and feed them. The grand edifices for the purpose scattered throughout the Union testify that it does, but a great fault to be found is in want of organization and concert of action between legislative bodies having these matters in charge. Large dormitories prevail in European asylums in preference to numbers of small rooms, because experience has demonstrated their usefulness in the prevention of suicides, the prompt care of the sick, and the enhanced ability of a few persons in watching a large number of patients. Dr. Spray has recently introduced this feature in the County Asylum, and the cost of construction of asylums is greatly lessened, but in very few places is it in vogue in this country. In England, the corridor is only found in the antiquated asylums, but it exists without exception in all asylums here. Statistics fully prove that the best results, both as to recoveries and expense, are obtainable from a number of small asylums, with a capacity not exceeding 700 inmates, rather than from larger asylums, but the lesson is unheeded with us. Medical treatment becomes less efficient to almost impossible the larger the asylum grows. In the European asylums, the history of each patient is carefully ascertained, and all ancestral peculiarities recorded. His condition and the treatment he undergoes are set down day by day till his death or discharge. With some noteworthy exceptions, this is not the rule in the United States. In many asylums, merely the name, age, dates of admission and discharge, only are recorded. A knowledge of the antecedents of a disease is so requisite to proper treatment, in the great majority of cases, as to make this neglect criminal, whether the fault of the State or of individuals. Across the sea, physicians are selected who have grown to eminence in the treatment of mental and nervous diseases, as officers for asylums. Treatment is based largely upon the conditions known to exist in the body of the patient; there are special forms in which, as yet, nothing but mitigating measures are justified, as in paretic dementia, which rarely, if ever, recovers, and while many other cases are hopeless, there are still many which become chronic only through failure to properly diagnose and institute prompt and suitable treatment. Many cases also recover where nothing has been done for them, and some even in spite of improper treatment, bnt this is no justification for withholding treatment. The same is true of all other diseases, and it is as necessary that the alienist should be well versed in his province of medicine, as any other specialist should be in his division, but special fitness is not a distinguishing characteristic of every American insane asylum physician, and if, through experience and much study, he has become an adept in this field, with corresponding loss of expertness in other branches of medicine, it often happens that in the rotation of office he must give place to some one else less versed in these matters, who must acquire his knowledge at the expense of many a patient's life.

An abstract of the methods used, and the principles governing the treatment of mental diseases, would be tedious reading to a popular audience. Suffice it to say that the bodily health must be maintained, and the circulation particularly regarded. Sometimes a slight scar will indicate a course of medicine the beneficial results of which may be predicted in nine cases out of ten. An injury to the head—frequently without visible evidence of it, and only the history of the case as a guide—may justify a surgical operation, in the happy issue of which our literature abounds. Correction of some physical or moral vice, and educational measures which call into requisition latent reasoning powers, are often successful. Male cases Nos. 250, 227, 289 at the County Asylum are good examples of recent cures, which are, and may be, effected wherever the proper interest in a case exists, with sufficient knowledge on the part of the medical attendant. We may well ask, what is the cause of so much want of progress in our psychiatrical affairs? Why is not legislation brought to bear upon the subject?

Those who believe that such things may be corrected by law would do well to consider an instance in point: The New York legislature had become fully impressed with the necessity for advancing the medical status of its asylums, and proceeded to equip one with all the paraphernalia of a pathological laboratory. About $50,000 was expended in this way, a liberal salary was fixed for a pathologist, who was finally selected. He had never been heard of before; he was and is ignorant of medical matters, and since his incumbency has published at an expense of $3,000 to the State plagiarised matter from the works of Rindfleisch, a German standard author on pathology.

The fault does not lie with legislatures, supervisors, superintendents, or the medical men of asylums that Europe is ahead of us in governmental fostering of such science. Some of the causes may be ascribable to conditions pointed out as likely to occur by Washington in his farewell address, but as this government is of the people and by the people, no one is to blame but the people themselves. A fountain cannot rise higher than its source. An aristocracy in Europe which, however, it may operate toward degrading the masses on the one hand, through its educational influences affiliates with and maintains fit men in professional State fields. While at home we escape the evil domination of monarchy, we cannot expect to find measures adopted and establishments reorganized on a basis which the people cannot understand.

So long as the populace know nothing of what has been accomplished by other nations in this field, so long will nothing be done to elevate the medical and scientific management of asylums.

As soon as the public grow to the requisite knowledge, an overturning of antiquated systems will proceed vigorously. The people of the State of Virginia, in 1882, were divided on a State debt issue. Among those who favored the payment of the debt were all the superintendents of asylums in the State, and they were summarily dismissed from office on the success of the repudiation measure. This was a popular judgment of unfitness to treat the insane. Herbert Spencer has said that America will give rise to the highest civilization the world has ever seen, nor need we think otherwise from the slow progress toward it in some directions. Changes for the better are being gradually made and will endure. Many fallacies have to be overcome, among them the ideas that it is better to neglect early treatment by delay in resorting to the asylum; that the stigma of insanity through asylum treatment is a greater calamity than the risk of perpetuating insanity through avoidance of asylum treatment; that jurors are more to be trusted than physicians, both as to knowledge of insanity and in matters requiring ordinary probity.

Society imposes a penalty upon efficiency in these abstract studies. If the student attend too closely to his work, his absorption and enthusiasm entail withdrawal from contact with the world at large, and its crude ideas as to how psychological problems should be dealt with. The world readily forgets those who are forgetting it, and however correct may be the student's methods, however conscientious and capable he may be in the performance of his duties, so long as the public is not educated up to his ideas he can expect no help from it. The general practitioner realizes that his chances for financial success are only through closing his books, forsaking his instruments of research, and cultivating so-called society, with its lap-dogs and small talk. A professional man is judged, as usually every one is judged, by the amount of money he obtains, notwithstanding the so well known fact that charlatanism invariably fattens where merit and skill may starve. As in every field of science, so it is in the psychiatrical, the world has advanced through the misunderstood, often reviled, efforts of its underpaid, neglected searchers after truth. Emotionalism demands the strangling of the living Jenner for proposing to make us first cousins to cows, and rushes to the apotheosis of the dead

Jenner for his having while living conquered a terrible disease.

The superstition which regarded insanity as a penalty for sins and teleological doctrines engendered fatalistic views of insanity, and hindered rational methods of research by ignorantly declaring mental processes insoluble.

The prevalence of purely subjective methods of study of mental phenomena perpetuated false views, and obstructed physiological method, even though *mens sana in corpore sano* had become cant. It is coming to be generally realized that the laws of the mind are part and parcel with the laws that govern the universe; that physics is more useful in psychology than metaphysics, and that mentality may not be understood without a knowledge of the anatomy and physiology of the brain, no more than the horologity of a clock may be grasped without knowledge of it mechanism.

But the laity cannot be blamed for want of information as to what has been accomplished toward clearing up these mysteries, while it is only within the past few years that these matters were taught in medical schools in America. Had there been as thorough instruction on these subjects in our medical schools, we would not see a dozen widely different forms of insanity sent to asylums as "softening of the brain." Some of the psychoses often erroneously included in this omnibus are curable.

The literature of alienistic knowledge is beginning to overflow from special repositories, such as the *Journal of Mental Science*, *Journal of Nervous and Mental Disease*, *Journal of Neurology and Psychiatry*, and *Alienist and Neurologist* into general medical periodicals. There is enough definiteness now about what is known of insanity to make it incumbent upon the general practitioner to apprise himself of what is known particularly as the family physician is usually the first one called in the incipiency of, and best time to properly treat, the mental complaint. It will not be necessary for him to dive into the minutiæ and polemics of psychiatry, but when asylum treatment is not available, problems as grave as are presented by great hæmorrhage, collapse, or pernicious fevers appear with the advent of mental derangement, and timely, judicious control may often restore reason.

So far as money can accomplish anything, it has been lavished by Europe upon psychiatrical problems. Vienna, Paris, London, Rome and Florence are prominent as centers of research. Nor has there been want of previous special training to fit the physicians there who lead in these matters. They are educated with special reference to physiological research. Not only the anatomy of man, but that of the lower animals is being worked out microscopically; the intricate mechanism of the nervous system is gradually being disentangled; the dynamics of life are being delved into, and many workers in special fields are contributing to synthetical knowledge, which is rapidly growing in exactness.

In subsidiary fields physiological chemistry has its Gamgee; biology its Huxley and Hæckel; minute anatomy its Meynert; vivisectional experimentation its Fritsch, Hitzig and Ferrier; pathology its Exner, as foremost representatives of a host of explorers, who work with scalpel, microscope, electrical apparatus and other scientific appliances year after year in the light afforded by Darwin and Spencer. Clinical observations are made by Hughlings-Jackson, Charcot, Maudsley, Brown-Sequard, and all the asylum officers in Europe, and a number of them on our side of the Atlantic. Among our native workers outside of asylums, we have Hammond, Spitzka, Kiernan, Mills, Wood, McBride, Schmidt, Jewell, Brower, Seguin, Morton, Hughes, and others.

As an illustration of one of the multitude of methods, Exner's process may be mentioned. From 2,000 cases of diseased conditions found in different parts of the brain, patiently and logically examined, in connection with the accompanying peculiarities exhibited by each patient before death, he has formulated laws which go to supplement the localization of function in the brain, and show us that while various parts of the brain are absolute centers for the movement of particular groups of bodily muscles, relative centers exist also for these same muscles in other parts of the brain. A flood of light was let in at once upon many apparently contradictory phenomena, and methods of observation have been correspondingly improved. The Italian Golgi, by an improved process of staining, has enabled us to trace out hitherto unrecognizable microscopic connections of nerve fibers. The importance of this may be understood by regarding the nerves as so many telegraph lines between certain stations, whose connection with each other must be ascertained before cause and effect can be affirmed. When the mechanism and the forces at work are both better understood, related phenomena are correspondingly better known.

The tendency of modern research is to show that the circulation of the blood plays a very important part in mental workings, and that upon the integrity of minute blood-vessels the mind depends for its existence, as much as upon the nerve cells and fibers the vascular system nourishes, and as Morel states, that "the brain is always the seat of the insanity, but not always the seat of its cause."

Some of the problems undergoing discussion by alienists are: Why are more of one nation than of another insane? What especially induces insanity? What temperaments are most predisposed? The precise defects in the organism which accompany insanity. How those predisposed to insanity may avoid it. The proper treatment of all kinds of insanity. How far treatment is serviceable. The prevention of insanity and its reduction to a minimum. These and kindred inquiries befit a social arrangement which, as Spencer says, survives only on condition that each generation of its members shall yield to the next benefits equivalent to those it has received from the last. The well being of each is involved in the well being of all. Whatever conduces to their vigor concerns you, for it diminishes the cost of everything you buy. Whatever conduces to their freedom from disease concerns you, for it diminishes your own liability to disease. Whatever raises their intelligence concerns you, for inconveniences are daily entailed on you by others' ignorance or folly. Whatever raises their moral characters concerns you, for at every turn you suffer from the average unconscientiousness."

To this end the medical and scientific standards of American asylums should be raised to that existing abroad. If there is justification in caring for and treating the insane at all, there is equal justification for the reduction of the study to a science that will tend toward obliterating as much insanity as lies in our power, and we may expect much from a people who work industriously in every field recognized as a paying one. It is as much the duty of a nation to explore the causes of diseased bodily conditions of its individuals, as it is to survey its lands geodetically or geologically, or with reference to its flora and fauna, and ultimately the information elicited by such explorations would be diffused among and benefit the .people. Our County Asylum, thanks to the intelligence of the Commissioners, ranks with the advancing condition of alienistic matters in the Union. Voluminous case books are being filled there day by day with the progress of each case, the results being communicated through medical journals, with pathological and microscopical details, in connection with all that can be learned concerning the patient. American asylums generally are gradually increasing their medical assistance.

The visiting physician system, as formerly organized, has proved valueless. The State should employ well recognized skilled specialists, in addition to the regular resident physicians. By recognition is meant repute among reputable physicians. What has the specialist accomplished? what does he know? should be the questions of fitness, and the superintendent of the asylum should be untrammeled in his selection and dismissal of these specialists. The same objections to having business interferences with the superintendent, apply with equal force to his contact with coadjutors.

The resident staff for a 500 population asylum should consist of medical superintendent, three assistant physicians, one pathologist, one female physician as gynæcologist, one clinical clerk. A consulting staff to visit the patients weekly, or as suggested by the superintendent, to consist of one specialist in skin diseases, one eye and ear physician, one surgeon. Internes could be selected from medical schools among those wishing to fit themselves in psychiatry, and the clinical clerk is in Europe selected on that basis. There need be no fear of increased expense in such an arrangement, for the consulting staff would serve for the honor of the positions, and internes receive no salaries from any hospital fund. The additional cost for board of the extra medical men could be afforded for ten years by one year's curtailment of unnecessary building details. Millions of dollars are expended in unwise architecture absolutely lost by the necessity for being undone in time. At Danvers, Massachusetts, a palace asylum was undergoing construction, while the insane of the State awaiting its completion starved and were frozen in dungeons of almshouses, and were without any medical attendance. This was before Gov. Butler's day. It is evident from such instances that mere desire for display was at the bottom of the erection of a grand asylum, and that no question of benefiting the prospective inmates by medical or any other treatment entered into the intentions of its founders. Can economy be said to have justified this condition of things?

The coming century will witness as great an advance in the methods of treating the insane as this century has surpassed the eighteenth in their treatment. The time is coming when, instead of millions being expended for palatial asylums, a few thousands will suffice for comfortable hospital construction, and the surplus be expended in curative measures, and in the search for the causes and the means for prevention of insanity. Asylum officials will be selected through special fitness for their vocations. Instead of its being as much as one's position was worth to ask for a post-mortem case of instruments, every hospital for the insane will become the nucleus of a busy faculty of investigators, and even six months work on the part of a competent physician will not be considered too much to devote to a single post-mortem investigation, with the occasional result of producing a Virchow, who could see more in a pathological specimen in one day than multitudes of other observers in a lifetime. The methods of research have been established, the course to pursue to arrive at definite results is now known, and the means and workmen are coming, for with the dawn of the twentieth century the exactness of the knowledge already evoked will have advanced by that time to an extent that will open the eyes of the meanest of the multitude to the necessity for more thorough investigation, not only of the brain, but of the entire body in connection with the *intra viam* history. There is nothing like the realization that a thing *pays* in America to induce activity.

"To tell whither a measure is drifting, it is important to take as data for sociological conclusions not the brief sequences, but the sequences that extend over centuries or are traceable throughout civilization," says Spencer. From brutality in the treatment of the insane in the past we have advanced to kindness, and the tendencies are toward a more careful study of the causes and means of prevention of insanity. The immediate effect being an amelioration of the condition of patients living, with the acquisition of ability to combat the causes of, and to prevent insanity in generations unborn.

---

ACTINOMYCOSIS DISCOVERED IN AMERICAN CATTLE.—Dr. William T. Belfield, of Chicago, has made the important discovery that actinomycosis exists in American cattle. He was asked by the Commissioner of Health of Chicago to investigate a disease in cattle which has generally been known as "swell-head," and has been called by veterinarians, Cancer, Sarcoma, etc. Five animals were examined by Dr. Belfield, and a very short study of the specimens under the microscope revealed the true nature of the disease. Actinomycosis was only recognized six weeks ago by Bollinger, of Munich, who announced that it was a parasitic disease due to the presence of a rapidly growing fungus. It has since been discovered in the hog and in man. It generally first attacks the jaws, and probably gains access to the deeper tissues through carious or defective teeth. It spreads into the tissues of the head, causing tumefactions, suppuration, finally, if unchecked, pyæmia, and death. It may gain the blood and be transferred to other parts of the body. This happens especially with man, upon whom the parasite acts most virulently. It is supposed that its source is the grain with which animals are fed. The disease is generally fatal, though prompt measures may check it. The meat of animals dying from actinomycosis is not of first quality. It is not, however, yet known that it is absolutely injurious. Thorough cooking, at any rate, destroys the parasite. Dr. Belfield's discovery is an important one; and should become promptly known to veterinarians and sanitary officials.—*New York Medical Record, Dec.* 8, 1883.

